# Nutritional Biomarker-Guided Prediction of Postoperative Pain Outcomes in Elderly Patients Using a Shapley Additive Explanations (SHAP)-Informed XGBoost Approach

**DOI:** 10.7759/cureus.85048

**Published:** 2025-05-29

**Authors:** Rafail Ioannidis, Despoina Sarridou, Adamantios Bampoulas, Christina Tsigalou, Pelagia Chloropoulou

**Affiliations:** 1 Anesthesiology and Pain Medicine, General Hospital of Drama, Democritus University of Thrace, Drama, GRC; 2 Anesthesiology, AHEPA University Hospital, Aristotle University of Thessaloniki, Thessaloniki, GRC; 3 Computer Science, University College Dublin, Dublin, IRL; 4 Medical Microbiology-Immunology, Democritus University of Thrace, Alexandroupolis, GRC; 5 Anesthesiology and Pain Medicine, Democritus University of Thrace, Alexandroupolis, GRC

**Keywords:** c-reactive proteins (crp), inflammation, mna-sf, nutric score, nutrition status, predict model, serum albumin, surgical pain

## Abstract

Nutritional status is increasingly recognized as a critical factor influencing perioperative outcomes. In elderly surgical patients, undernutrition and inflammation may play a key role in the development of both acute and chronic postoperative pain. Despite this, few studies have explored their predictive value using machine learning approaches. This prospective, non-interventional study aimed to assess whether nutritional screening tools and biochemical biomarkers could predict postoperative pain trajectories, both acute and chronic, in elderly surgical patients, using interpretable machine learning methods. A total of 108 patients aged ≥70 undergoing elective surgery under spinal or general anesthesia were enrolled. Preoperative assessments included the Mini Nutritional Assessment-Short Form (MNA-SF), the modified Nutritional Risk in the Critically Ill score (mNUTRIC), the Acute Physiology and Chronic Health Evaluation (APACHE), the Sequential Organ Failure Assessment (SOFA), red cell distribution width (RDW), C-reactive protein (CRP), serum bilirubin, serum albumin, serum calcium, and serum ferritin. Pain was recorded at four time points: pre-surgery, immediately post-surgery, 30 days, and six months. XGBoost classifiers were trained to predict pain at each time point, using a custom ordinal-aware loss function and evaluated via accuracy, F1-scores, and confusion matrices. Feature importance was analyzed with SHAP values for model interpretability. The results were the following: Predictive accuracy varied across timepoints: 36% (pre-surgery), 52% (acute post-surgery), 42% (30 days), and 55% (six months). Misclassifications were predominantly within one ordinal level of the true pain score. SHapley Additive exPlanations (SHAP) analysis revealed APACHE, CRP, albumin, ferritin, and MNA-SF as key predictors across models. Chronic pain predictions at six months showed the highest accuracy and stability, highlighting the relevance of preoperative nutritional and inflammatory markers in long-term pain outcomes. In conclusion, this study uses interpretable machine learning in an innovative way to link nutritional screening and inflammation markers to postoperative pain in elderly patients. The findings emphasize the predictive role of nutritional and inflammatory status in pain trajectories and suggest that integrating such assessments into perioperative care may improve personalized pain management and recovery outcomes in the elderly.

## Introduction

Nutritional screening has become an increasingly important focus in perioperative medicine. Anesthesiologists should be vigilant about patients' nutritional status, recognizing the potential risks of undernutrition or malnutrition and being proficient in assessing nutrition both before and after surgery. However, standardized definitions for undernutrition and malnutrition are still evolving [1]. Nutritional status is closely associated with various complications in the perioperative and postoperative periods [2]. Among these, postoperative pain stands out as a major concern, not only for anesthesiologists but also for patients. Optimal pain management following surgery is essential to support recovery and ensure a smooth return to normal daily activities. Moreover, effective control of postoperative pain is crucial in preventing the transition from acute to chronic pain [3]. Currently, there is limited evidence in the literature on the relationship between undernutrition and either acute or chronic pain [4,5]. Nevertheless, it is worth exploring whether biomarkers used to detect undernutrition, along with widely applied nutritional assessment tools, are associated with increased pain scores during these periods [6,7].

The Mini Nutritional Assessment (MNA) is a validated and practical tool for identifying nutritional risk, particularly among elderly patients, whether living independently or in clinical settings [8,9]. It encompasses questions related to nutrition, general health, functional independence, quality of life, cognition, mobility, and self-perceived health status, all tailored for the geriatric population [10]. The European Society for Clinical Nutrition and Metabolism (ESPEN) endorses the MNA for routine use in geriatric assessments [11]. To improve practicality, Rubenstein et al. introduced the MNA Short-Form (MNA-SF), which comprises six key questions selected from the full version based on their high sensitivity, specificity, and correlation with overall assessment outcomes [12]. This abbreviated form categorizes individuals as either well-nourished or at risk of malnutrition, with further assessment using the full MNA warranted only if risk is identified. The MNA-SF has demonstrated comparable diagnostic accuracy to the full MNA while offering a more time-efficient approach [13,14].

The Nutrition Risk in Critically Ill (NUTRIC) score, created by Heyland et al. [15], is designed to identify ICU patients who are likely to benefit from aggressive protein-energy nutritional therapy [16,17]. It incorporates baseline clinical factors to evaluate how nutrition interventions might influence patient outcomes during critical illness [18-20]. While interleukin-6 (IL-6) is included in the original NUTRIC score, its limited availability in many Intensive Care Units (ICUs) has led to the development of the modified NUTRIC (mNUTRIC) score, which omits IL-6. The mNUTRIC score assesses seven clinical and laboratory parameters: age, Body Mass Index (BMI), Acute Physiology and Chronic Health Evaluation II (APACHE) score, serum albumin, creatinine, sodium, and glucose levels. Research indicates that an mNUTRIC score of four or more is associated with increased mortality risk in critically ill patients. This tool not only helps identify patients who may benefit from targeted nutritional support but also aids clinicians in making informed nutritional decisions. Importantly, the mNUTRIC score can be applied effectively in the perioperative setting without modification [21,22].

The main objective of this non-interventional, observational, prospective study is to evaluate the significance of specific biomarkers and nutritional screening tests as prediction tools for the level of postoperative pain, whether acute or chronic, in surgical elderly patients with or without nutritional deficiencies to maximize postoperative care, address knowledge gaps and personalize treatment focusing on improving both pain management and nutritional support for elderly surgical patients-ultimately enhancing their recovery and overall quality of life.

## Materials and methods

Ethics approval for this study was granted by the Ethics Committee of the Medical School at Democritus University of Thrace (DUTh), Greece, under registration number ΔΠΘ/ΤΙΑΤΡ/35548/3207, dated February 27, 2020. The study protocol was registered on ClinicalTrials.gov with the identifier NCT06802575.

This prospective, observational, non-interventional study included 108 elderly surgical patients aged 70 years and older, regardless of their nutritional status, and was conducted over a four-year period from April 2020 to March 2024. All participants provided informed consent, which included both a comprehensive study-specific consent form and the hospital’s standard consent form for anesthesia and surgical procedures. Participants were fully informed about the study objectives and procedures.

Exclusion criteria comprised individuals with psychiatric disorders, cancer, those receiving corticosteroid therapy or chemotherapy, patients undergoing cardiothoracic surgery, and individuals with chronic inflammatory gastrointestinal conditions. These are categories of patients with a high likelihood of being malnourished from a pre-existing disease without a real connection to the surgical pathology. 

The weight of every single patient was monitored pre-operatively to calculate exactly the BMI of each patient. All enrolled patients received either spinal or general anesthesia. Prior to anesthesia induction, standard monitoring was implemented, including electrocardiography, pulse oximetry, and non-invasive blood pressure measurement. For cases requiring general anesthesia, additional monitoring included bispectral index (BIS), nociception level (NOL), and core temperature. A uniform anesthetic protocol was applied to all patients, utilizing the same drug regimen and a consistent type of atraumatic 25G spinal needle (Pencan) for spinal blocks. Also, throughout the duration of the patient’s stay in the hospital, there was a daily calculation of the patient’s weight to avoid possible loss in BMI. 

This study employed a supervised machine learning approach to model and predict postoperative pain trajectories using structured clinical data. The dataset consisted of collected preoperative and postoperative patient metrics, derived from surgical cases. The primary outcomes were pain scores recorded at four clinically relevant time points: before surgery (pre-surgery pain), immediately after surgery (acute post-surgery pain), 30 days postoperatively, and six months postoperatively (chronic pain). Each pain outcome was modelled independently using the same set of clinical predictors.

Ten predictor variables were selected and conducted preoperatively based on clinical relevance and literature-supported associations with surgical risk and recovery. These included the MNA-SF, the mNUTRIC, the APACHE, the Sequential Organ Failure Assessment (SOFA), red cell distribution width (RDW), C-reactive protein (CRP), serum bilirubin, serum albumin, serum calcium, and serum ferritin. All variables were numeric and collected from laboratory or clinical scoring systems within the perioperative period. The outcome variables were pain scores, assessed on a discrete ordinal scale at the four designated time points. For modelling purposes, each pain score was treated as an ordinal integer. Label encoding was used to convert these scores to a zero-based format suitable for classification modelling. The ordinal nature of the pain levels was preserved throughout the modelling pipeline.

The dataset was first screened for class imbalance across the outcome categories. If all classes contained at least two samples, a stratified train-test split was used to maintain proportional representation in both training and testing sets. If rare classes (fewer than two samples) were detected, a non-stratified 70:30 split was applied. Class imbalance was further addressed using sample weights, which were computed using a class frequency-based weighting scheme function from the scikit-learn library. This ensured that rarer pain scores had proportionally greater influence on model training. All features were included in their raw form, without normalisation or scaling, given the tree-based nature of the model, which is invariant to feature scaling. No imputation was necessary, as the dataset was complete.

XGBoost classification was used to model each pain outcome independently. The objective function was set to multi:softprob to allow the model to output class probabilities rather than hard labels, which enabled greater flexibility in evaluating ordinal misclassifications. The model was optimized using randomized hyperparameter search with three-fold cross-validation. Key hyperparameters tuned included the number of trees, maximum tree depth, learning rate, subsample ratio, and column subsample ratio per tree.

The optimisation criterion was not standard classification accuracy or log-loss. Instead, a custom ordinal-aware loss function was implemented using a distance-aware penalty. This function applied a penalty of zero when the predicted class matched the true class, a penalty of one when the prediction differed by one ordinal level, and a penalty equal to the difference minus one when the prediction error exceeded a single level. This design reflects the clinical reality that minor deviations in pain classification are tolerable, whereas larger errors are more consequential. Each model was evaluated using traditional classification metrics, including overall accuracy, macro- and weighted-averaged precision, recall, and F1-score. Confusion matrices were generated to visualise prediction distributions, and particular attention was given to whether misclassifications were ordinally close to the true labels. This evaluation allowed for qualitative assessment of model consistency and the practical impact of prediction errors.

To interpret the contribution of individual predictors to the output of each model, SHapley Additive exPlanations (SHAP) values were computed using the TreeExplainer algorithm. SHAP values quantify the marginal contribution of each feature to the prediction for each sample. Global feature importance was assessed by calculating the mean absolute SHAP value across all test set predictions. Bar plots of feature importance were generated to highlight the most influential clinical variables for each pain outcome. This explainability step was essential in connecting the machine learning model to the underlying clinical mechanisms. By identifying features with consistent influence across outcomes, the SHAP analysis provided interpretable insight into the physiological and biochemical drivers of pain severity. All analyses were conducted using Python 3.9, and the scikit-learn library on an AMD Ryzen 5 8640HS with Radeon 760M Graphics 3.50 GHz system with 16 GB RAM running Windows 11 operating system.

## Results

Predictive accuracy and confusion matrices

The predictive performance of the XGBoost classifier varied across the four pain outcomes, as visualised by the confusion matrices below. Each confusion matrix illustrates the frequency of predicted versus actual pain classes, with darker shades of blue indicating a higher frequency of predictions within each cell. Accurate predictions concentrate along the diagonal, representing exact matches between predicted and observed pain scores. Given the ordinal nature of pain scales, predictions near the diagonal-where the predicted and actual scores differ by only one class-represent clinically minor misclassifications.

**Figure 1:**
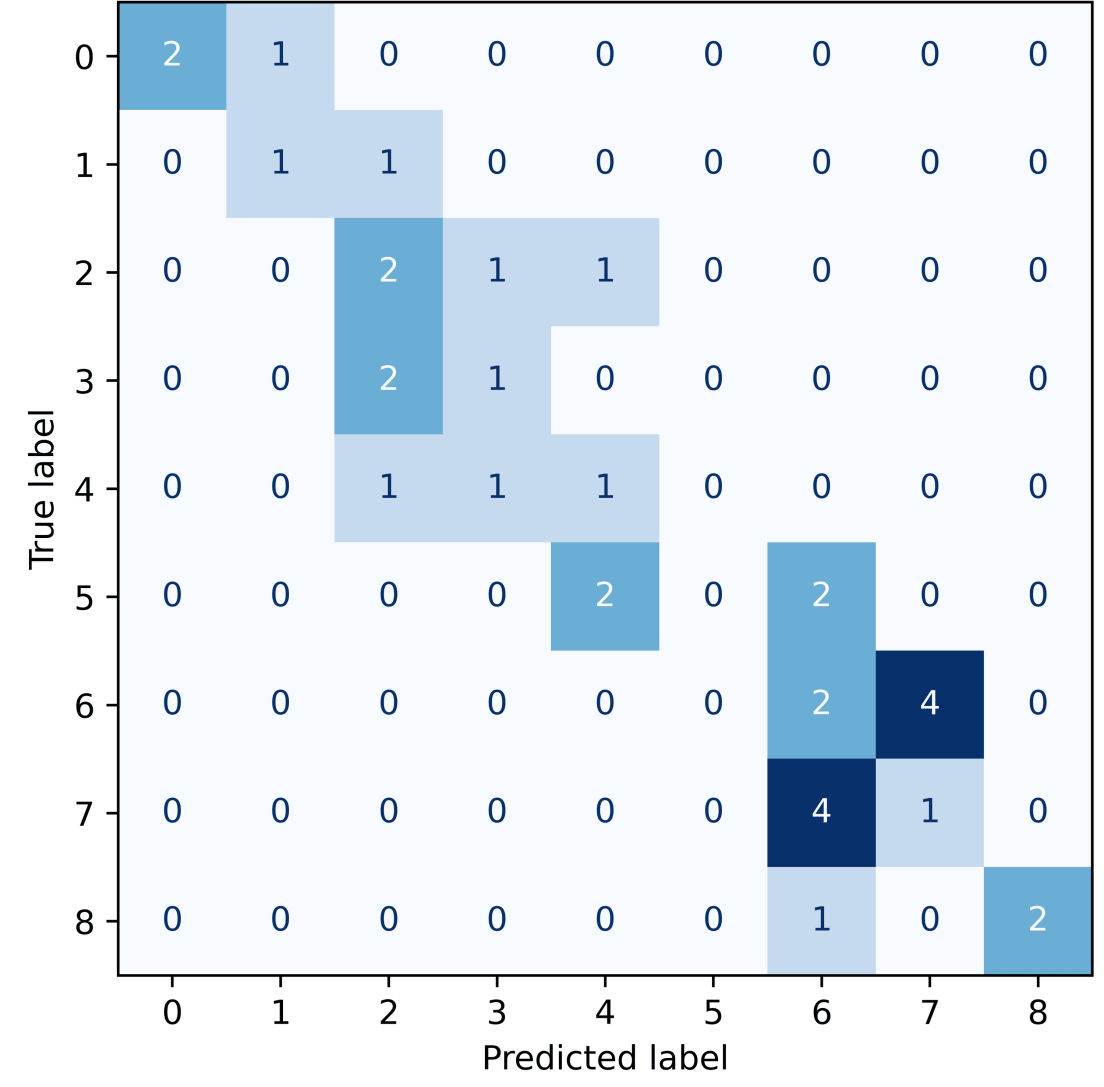
Confusion matrix for pre-surgery pain predictions using the XGBoost classifier. Confusion matrix illustrating the accuracy of predicted pre-surgery pain scores relative to true labels. Misclassifications are primarily confined to adjacent pain score levels (e.g., 6↔7, 2↔3), which is acceptable given the subjective nature of pain. Accuracy for more extreme scores (e.g., 0 or 8) is relatively strong.

For pre-surgery pain (Figure 1), the confusion matrix indicates moderate predictive accuracy (36%), with many misclassifications remaining adjacent to the true class, thus reflecting minor clinical deviations.

**Figure 2:**
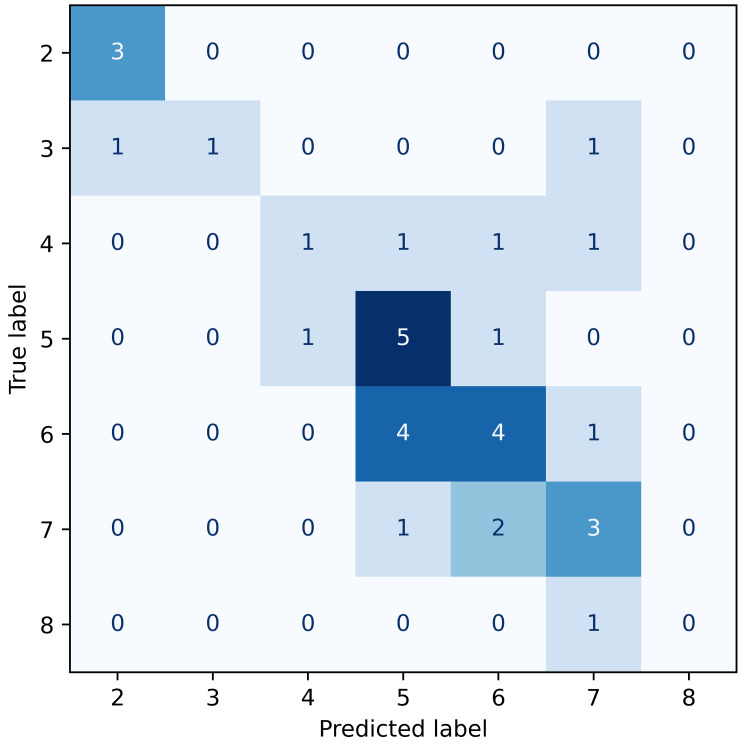
Confusion matrix for acute post-surgery pain predictions. Confusion matrix illustrating the accuracy of predicted acute post-surgery pain scores relative to true labels. Classifications for scores 5 and 6 are relatively accurate, but there is some confusion between classes 6 and 7, indicating the model has difficulty distinguishing between closely related but higher pain scores.

Acute post-surgery pain predictions (Figure 2) demonstrated improved accuracy (52%), with a clearer diagonal dominance, indicating better reliability of predictions immediately following surgery.

**Figure 3:**
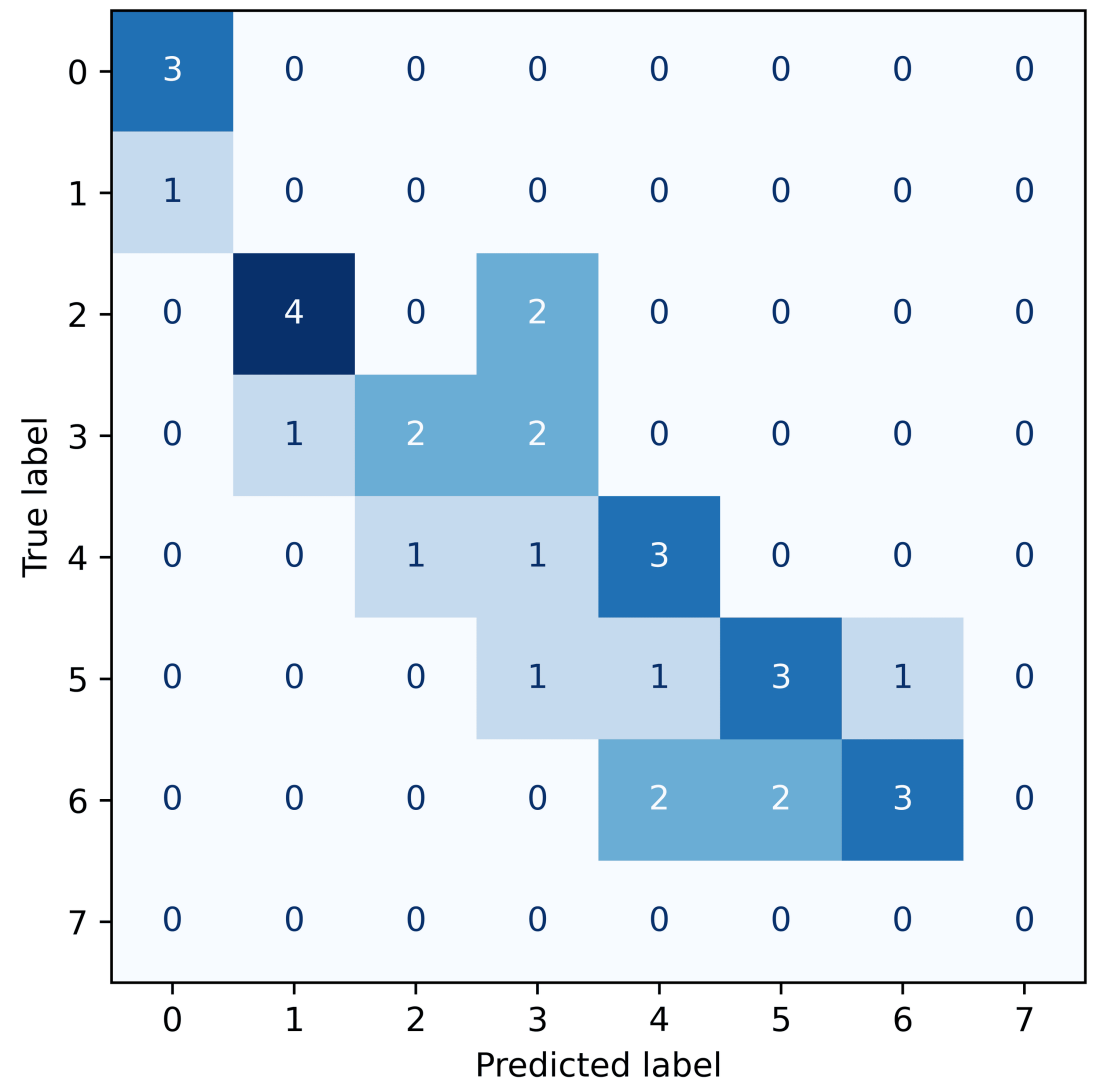
Confusion matrix for pain at 30 days post-surgery. Confusion matrix illustrating the accuracy of predicted pain outcomes at 30 days relative to true labels. The model performs reasonably well for mid-range pain scores (e.g., 4–6), with common errors involving predictions that are one point away from the true score — indicating acceptable clinical tolerance for ordinal classification.

The model predicting pain at 30 days post-surgery (Figure 3) displayed a moderate accuracy of 42%, with frequent minor misclassifications near the diagonal.

**Figure 4:**
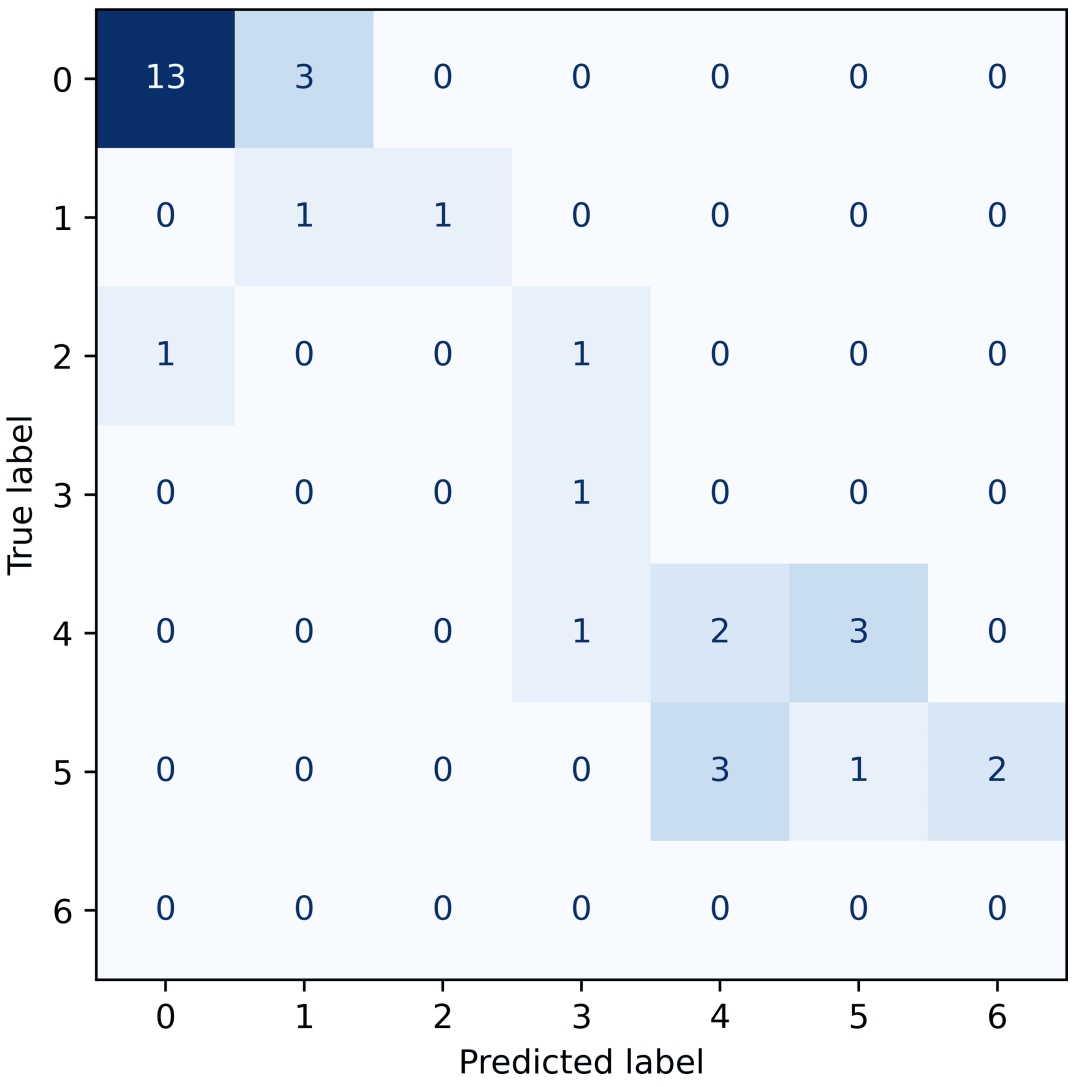
Confusion matrix for chronic pain at six months. Confusion matrix showing the distribution of true versus predicted pain scores at six months. Most predictions cluster along the diagonal, especially for the most frequent class (score = 0), indicating generally good classification performance. However, some misclassifications are present between adjacent classes (e.g., 0→1, 4→5), which are less concerning given the ordinal nature of pain scores.

Chronic pain at six months (Figure 4) showed the highest predictive accuracy (55%), evident by a more defined diagonal distribution of correct predictions and fewer substantial errors, indicating effective long-term pain classification.

To summarize and compare model performance across the four pain outcomes, Table 1 presents the classification accuracy alongside macro- and weighted-averaged precision, recall, and F1-scores. While accuracy alone does not fully capture model reliability for ordinal classification, the macro and weighted metrics offer additional perspectives on class-level performance and class balance sensitivity. As shown, the model performed best in predicting chronic pain at six months, with the highest overall accuracy and weighted F1-score, suggesting greater model stability in long-term outcomes. In contrast, pre-surgery pain predictions had the lowest accuracy and F1 metrics, possibly reflecting variability in subjective pain perception or limited predictive signal from preoperative features.

**Table 1:** Overall Classification Metrics by Pain Outcome

Outcome	Accuracy	Macro Precision	Macro Recall	Macro F1	Weighted Precision	Weighted Recall	Weighted F1
Pre-surgery pain	0.36	0.43	0.39	0.4	0.38	0.36	0.36
Acute post-surgery pain	0.52	0.52	0.46	0.45	0.53	0.52	0.49
Pain at 30 days post-surgery	0.42	0.42	0.42	0.41	0.46	0.42	0.43
Chronic pain at six months	0.55	0.31	0.4	0.32	0.59	0.55	0.56

SHAP feature importance and color-coded class contributions

The images below display the SHAP plots, which quantify the importance of clinical features in predicting pain outcomes at each time interval. SHAP values represent the mean absolute contribution of each predictor to the model predictions across all samples, facilitating an interpretable ranking of features. In these figures, different colours correspond to different pain categories (ordinal pain scores), highlighting how individual predictors contribute variably across different severity categories.

**Figure 5:**
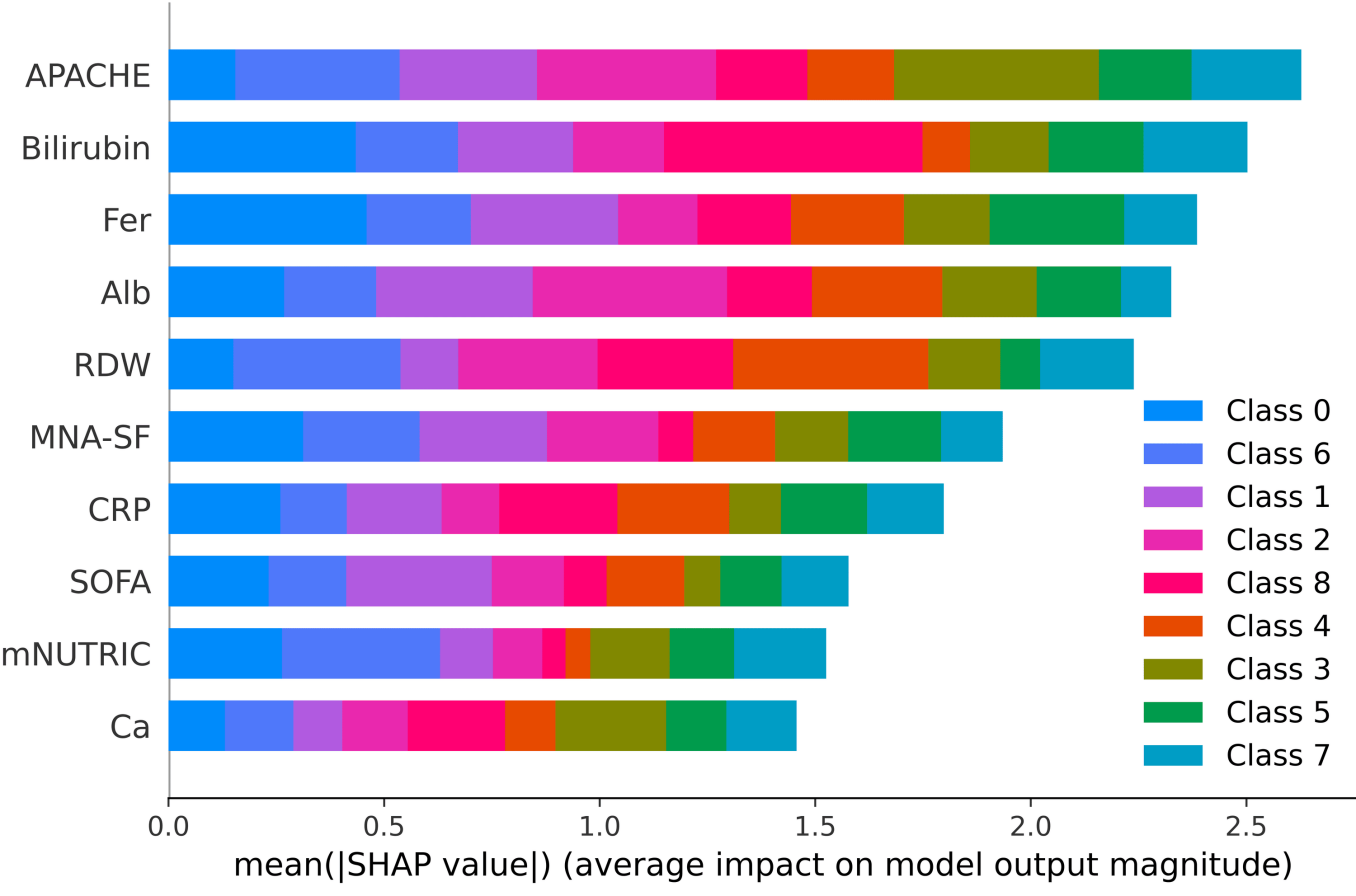
SHapley Additive exPlanations (SHAP) feature importance plot for pre-surgery pain predictions. SHAP bar plot representing the importance of clinical features in predicting pre-surgery pain levels. Segment colours indicate the share of importance attributed to each class (pain score) with APACHE and bilirubin appearing dominant in predicting baseline pain levels. APACHE: Acute Physiology and Chronic Health Evaluation Bilirubin: Serum bilirubin Fer: Serum ferritin Alb: Serum albumin RDW: Red cell distribution width MNA-SF: Mini Nutritional Assessment–Short Form CRP: C-reactive protein SOFA: Sequential Organ Failure Assessment mNUTRIC: modified Nutritional Risk in the Critically Ill score Ca: Serum calcium

For pre-surgery pain (Figure 5), SHAP analysis identified APACHE, bilirubin, ferritin, and albumin as critical predictors. The colour coding reveals that APACHE contributes substantially to both lower (e.g., classes 0 and 1, depicted in blue and purple shades) and higher pain categories (e.g., class 8, depicted in pink), reflecting its broad predictive relevance across the entire pain spectrum.

**Figure 6:**
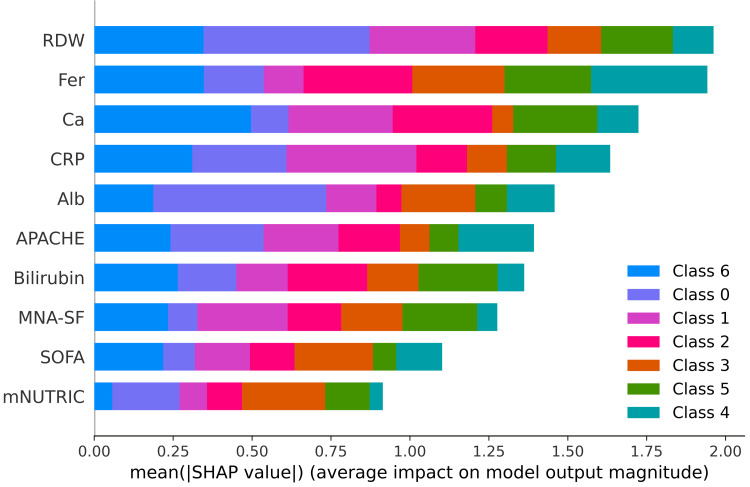
SHapley Additive exPlanations (SHAP) feature importance plot for acute post-surgery pain. Mean SHAP values for features used in the classification of acute post-surgery pain. Bar lengths indicate overall impact on model predictions, with color denoting the contribution per class (pain score) with RDW and Feritin levels being the most influential features. APACHE: Acute Physiology and Chronic Health Evaluation Bilirubin: Serum bilirubin Fer: Serum ferritin Alb: Serum albumin RDW: Red cell distribution width MNA-SF: Mini Nutritional Assessment–Short Form CRP: C-reactive protein SOFA: Sequential Organ Failure Assessment mNUTRIC: modified Nutritional Risk in the Critically Ill score Ca: Serum calcium

In acute post-surgery pain predictions (Figure 6), RDW, ferritin, calcium, and CRP emerged as prominent contributors. In this case, distinct colours highlight their differential predictive impact on varying pain severity classes - for instance, RDW significantly influences the prediction of higher pain categories (e.g., classes 4 and 5, shown in teal and green), indicating its particular relevance to more severe acute postoperative pain.

**Figure 7:**
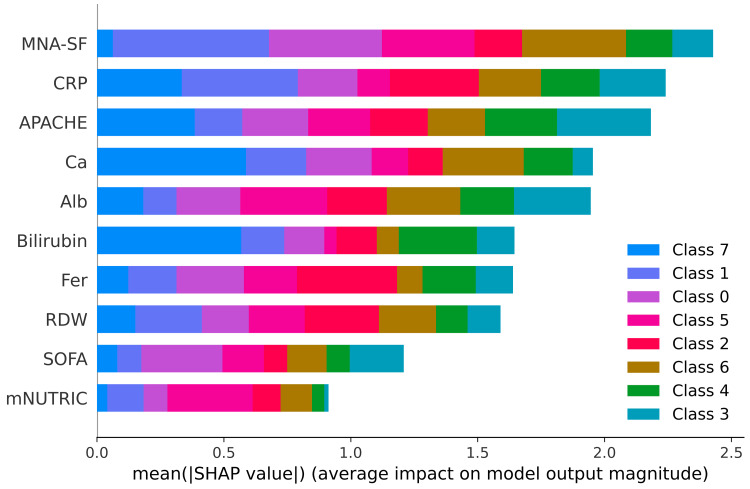
SHapley Additive exPlanations (SHAP) feature importance plot for pain at 30 days post-surgery. SHAP summary plot displaying the global feature importance for the XGBoost model predicting pain scores at 30 days post-surgery. Each bar represents the mean SHAP magnitude, with colour coding by pain score class. MNA-SF and CRP are the most significant predictors for pain at 30 days, with APACHE also contributing meaningfully. The relatively even distribution of feature contributions across multiple classes suggests a more complex and distributed pattern of importance. APACHE: Acute Physiology and Chronic Health Evaluation Bilirubin: Serum bilirubin Fer: Serum ferritin Alb: Serum albumin RDW: Red cell distribution width MNA-SF: Mini Nutritional Assessment–Short Form CRP: C-reactive protein SOFA: Sequential Organ Failure Assessment mNUTRIC: modified Nutritional Risk in the Critically Ill score Ca: Serum calcium

For pain at 30 days post-surgery (Figure 7), MNA-SF, CRP, APACHE, and calcium were influential predictors. The SHAP colour coding reveals nuanced patterns: for example, MNA-SF strongly influences the prediction of the most severe pain categories (depicted in green and teal), emphasising the clinical relevance of nutritional status in persistent postoperative pain.

**Figure 8:**
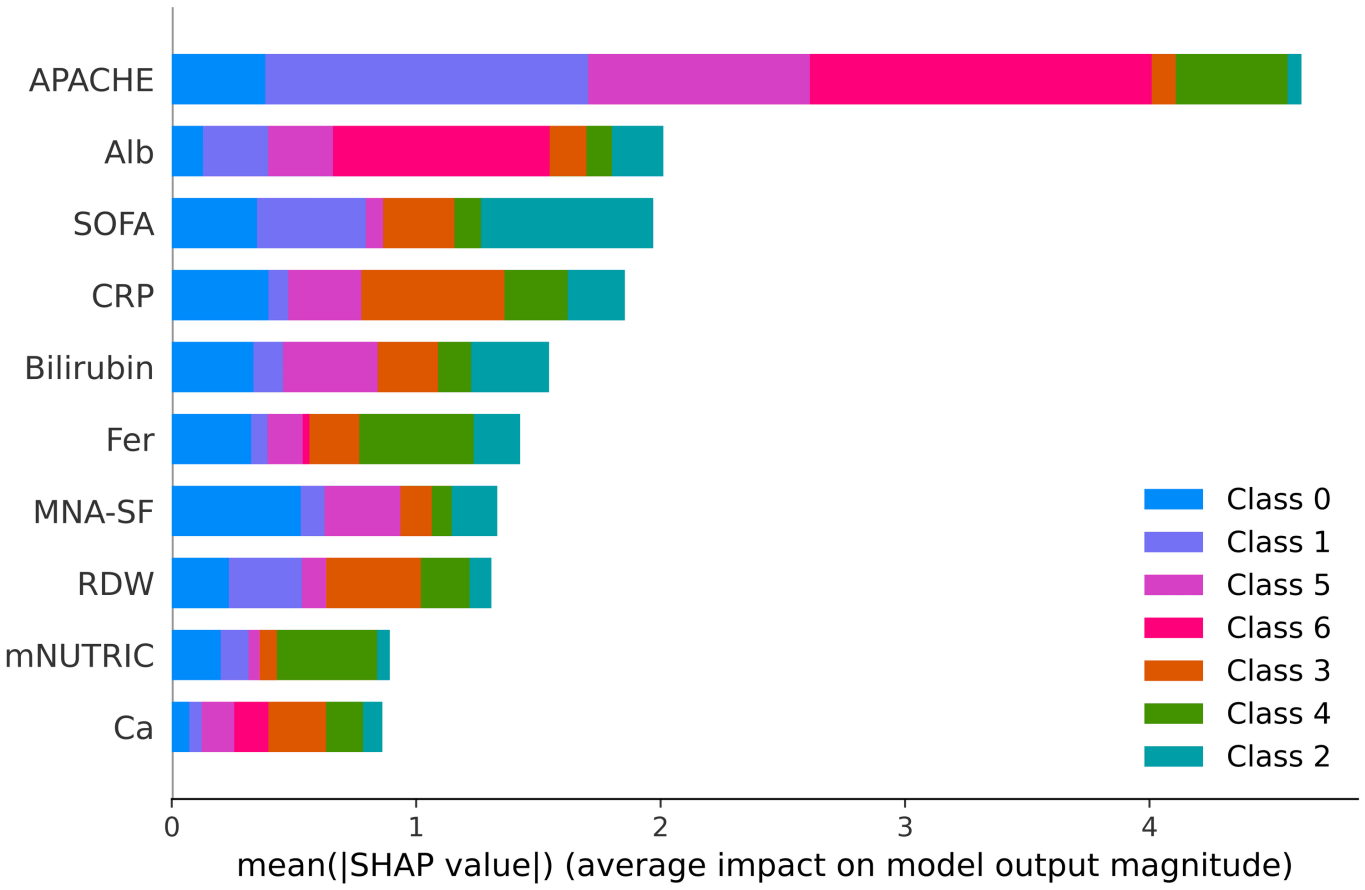
SHapley Additive exPlanations (SHAP) feature importance plot for chronic pain at six months. Mean absolute SHAP values indicating the relative contribution of each feature to the XGBoost model predicting chronic pain scores at six months. Colour segments represent the distribution of SHAP values across individual pain score classes. The APACHE score is by far the most influential predictor of chronic pain outcomes, followed by albumin and SOFA scores. APACHE: Acute Physiology and Chronic Health Evaluation Bilirubin: Serum bilirubin Fer: Serum ferritin Alb: Serum albumin RDW: Red cell distribution width MNA-SF: Mini Nutritional Assessment–Short Form CRP: C-reactive protein SOFA: Sequential Organ Failure Assessment mNUTRIC: modified Nutritional Risk in the Critically Ill score Ca: Serum calcium

Considering the prediction of chronic pain at six months (Figure 8), SHAP analysis shows the importance of APACHE, albumin, SOFA, and CRP. The different colours indicate how APACHE contributes prominently to the prediction of both low (blue, purple) and high pain categories (pink, red), emphasising its predictive versatility. CRP and albumin similarly show distinct colour patterns, indicating their differential impact on various chronic pain severity categories.

## Discussion

The machine learning model performed best in our study in predicting chronic pain at six months, suggesting greater model stability in long-term outcomes. Regarding the SHAP values for pre-surgery pain, APACHE, bilirubin, ferritin and albumin were identified as key predictors. In acute post-surgery pain RDW, ferritin, calcium and CRP showed critical prediction capability. For pain at 30 days post-surgery, MNA-SF, CRP, APACHE and calcium emerged as key predictors. Last but not least, APACHE, albumin, SOFA and CRP were identified as critical predictors for chronic pain at six months. From a clinical standpoint, this study’s findings suggest that nutritional status, inflammation, and illness severity should not be viewed in isolation. Instead, they are part of a broader constellation of interrelated factors that significantly influence postoperative outcomes. There is an observed correlation between pain at 30 days post-surgery and the development of chronic pain at six months, indicating that early postoperative complications can predict a high risk of persistent pain. This underscores the need for comprehensive preoperative evaluations that assess multiple dimensions of patient health.

Also, the predictive performance of the XGBoost classifier across different postoperative pain outcomes presents a compelling case for the integration of machine learning in perioperative risk assessment and pain management. Model accuracy varied depending on the pain timeframe, with performance improving progressively from preoperative to chronic postoperative phases. The model achieved moderate accuracy for pre-surgery and 30-day postoperative pain outcomes, and higher accuracy for acute post-surgery and chronic pain at six months. These trends suggest that while immediate pain responses may involve unpredictable intraoperative or individual factors, long-term pain outcomes are more closely linked to measurable preoperative and perioperative clinical variables. Importantly, most misclassifications occurred within one class of the true label, which represents minimal clinical deviation given the ordinal structure of pain scales. This proximity of predicted to actual scores supports the model's utility for real-world clinical application, particularly for risk stratification rather than precise point estimation. It is very important for the value of our study to underline how innovative it is, because it integrates machine learning for the evaluation of the significance of specific biomarkers and nutritional screening tests as prediction tools for the level of postoperative pain, whether acute or chronic, in surgical elderly patients with or without nutritional deficiencies. To compare model performance across the four pain outcomes, Table 1 presents the classification accuracy alongside macro- and weighted-averaged precision, recall, and F1-scores. While accuracy alone does not fully capture model reliability for ordinal classification, the macro and weighted metrics offer additional perspective on class-level performance and class balance sensitivity. As shown, the model performed best in predicting chronic pain at six months, with the highest overall accuracy and weighted F1-score, suggesting greater model stability in long-term outcomes. In contrast, pre-surgery pain predictions had the lowest accuracy and F1 metrics, possibly reflecting variability in subjective pain perception or limited predictive signal from preoperative features. The confusion matrices and SHAP analyses offer a comprehensive and clinically interpretable picture of model performance. While overall predictive accuracy varied across the various pain types, confusion matrices illustrate that most errors were minor, with predictions typically adjacent to true classes. The SHAP plots, with their colour-coded representations, provide detailed insights into how clinical predictors differentially influence pain across severity levels. The consistent prominence of inflammation-related markers (C-reactive protein, ferritin), nutritional factors (albumin, MNA-SF), and illness severity indicators (APACHE, SOFA) across all outcomes reinforces their clinical significance, highlighting targets for potential intervention. These findings demonstrate the utility of integrating interpretable machine learning methods into clinical decision-making, providing valuable guidance for personalised pain management strategies in perioperative care.

Notably, no previous studies in the current medical literature have specifically examined the combined relationship between nutritional status, inflammation, and both acute and chronic postoperative pain. This absence highlights the novelty and significance of the present study’s findings. To date, limited data exist linking malnutrition and pain, primarily in relation to specific conditions such as chronic musculoskeletal pain. For instance, Komolsuradej et al. demonstrated a positive association between nutritional deficiencies and musculoskeletal pain using the MNA-SF [23], while Elma et al.’s systematic review identified connections between dietary habits and conditions like osteoarthritis and fibromyalgia [24].

The APACHE and SOFA scores, widely validated as measures of disease severity and predictors of mortality, also emerged as relevant tools for predicting postoperative pain. These scores appear to bridge the relationship between illness severity, pain perception, and nutritional status [25,26]. A particularly intriguing finding of the study is that none of the examined input variables demonstrated exceptionally predictive value for short-term pain outcomes. This may be attributed to the consistent use of effective anesthetic techniques, which ensured adequate immediate postoperative analgesia - a core element of anesthetic practice. Moreover, recent literature has highlighted the anesthesiologist's evolving role in perioperative nutritional management, reinforcing the importance of selecting key preoperative markers to assess nutritional risk [27]. Existing literature confirms that both malnutrition and inflammation contribute to decreased serum albumin levels. Inflammation reduces albumin synthesis while increasing its fractional catabolic rate, and in severe cases, shifts albumin out of the vascular compartment. This creates a detrimental cycle where inflammation leads to anorexia, hampers protein and energy utilization, and accelerates albumin degradation [28-30].

Despite the novelty of our study, it has some limitations. One limitation is the inclusion of various types of surgical procedures from different surgical subspecialties. Although we excluded cardiothoracic and cancer surgeries and had a large patient sample, focusing on a single surgical subspecialty in future studies, such as orthopedic surgery, could provide more targeted insights. Another limitation is the inclusion of patients who received both general and regional anesthesia. Still there is no consensus regarding their impact of postoperative recovery and morbidity in the elderly population and it is still a matter of debate. The choice of providing general or regional anesthesia to an elder patient is multifactorial and also time sensitive.

## Conclusions

In summary, the analysis of confusion matrices and SHAP plots illustrates that the XGBoost classifier provides clinically meaningful predictions of perioperative pain outcomes. Notably, predictive accuracy improved over time, with the highest performance observed in forecasting chronic pain at six months post-surgery. This trend suggests that long-term pain outcomes may be more strongly associated with measurable preoperative factors, making them more amenable to accurate prediction through machine learning models. The SHAP analyses supported further the consistent importance of inflammation markers (CRP, ferritin), nutritional indicators (albumin, MNA-SF), and illness severity scores (APACHE, SOFA), underscoring their relevance in shaping postoperative pain trajectories. These results not only validate the predictive model but also point toward actionable clinical targets for early intervention. Collectively, this approach demonstrates the value of combining advanced machine learning with explainable analytics to support individualized pain management and improve outcomes for elderly surgical patients.
